# Tumor Growth Suppression Induced by Biomimetic Silk Fibroin Hydrogels

**DOI:** 10.1038/srep31037

**Published:** 2016-08-03

**Authors:** Le-Ping Yan, Joana Silva-Correia, Viviana P. Ribeiro, Vera Miranda-Gonçalves, Cristina Correia, Alain da Silva Morais, Rui A. Sousa, Rui M. Reis, Ana L. Oliveira, Joaquim M. Oliveira, Rui L. Reis

**Affiliations:** 1 3B’s Research Group–Biomaterials, Biodegradables and Biomimetics, University of Minho, Headquarters of the European Institute of Excellence on Tissue Engineering and Regenerative Medicine, AvePark, Parque de Ciência e Tecnologia, Zona Industrial da Gandra, 4805-017 Barco, Guimarães, Portugal; 2 ICVS/3B’s–PT Government Associate Laboratory, Braga/Guimarães, Portugal; 3 Life and Health Science Research Institute (ICVS), School of Health Sciences, University of Minho, Campus de Gualtar, 4710-057 Braga, Portugal; 4 Molecular Oncology Research Center, Barretos Cancer Hospital, Barretos, São Paulo, Brazil; 5 CBQF–Center for Biotechnology and Fine Chemistry, School of Biotechnology, Portuguese Catholic University, Porto, 4200 – 072, Portugal

## Abstract

Protein-based hydrogels with distinct conformations which enable encapsulation or differentiation of cells are of great interest in 3D cancer research models. Conformational changes may cause macroscopic shifts in the hydrogels, allowing for its use as biosensors and drug carriers. In depth knowledge on how 3D conformational changes in proteins may affect cell fate and tumor formation is required. Thus, this study reports an enzymatically crosslinked silk fibroin (SF) hydrogel system that can undergo intrinsic conformation changes from random coil to β-sheet conformation. In random coil status, the SF hydrogels are transparent, elastic, and present ionic strength and pH stimuli-responses. The random coil hydrogels become β-sheet conformation after 10 days *in vitro* incubation and 14 days *in vivo* subcutaneous implantation in rat. When encapsulated with ATDC-5 cells, the random coil SF hydrogel promotes cell survival up to 7 days, whereas the subsequent β-sheet transition induces cell apoptosis *in vitro*. HeLa cells are further incorporated in SF hydrogels and the constructs are investigated *in vitro* and in an *in vivo* chick chorioallantoic membrane model for tumor formation. *In vivo*, Angiogenesis and tumor formation are suppressed in SF hydrogels. Therefore, these hydrogels provide new insights for cancer research and uses of biomaterials.

Hydrogels have been extensively studied in tissue engineering and regenerative medicine as they may resemblance the extracellular matrix microenvironment^1^,^2^. Harnessing the properties of hydrogels is vital to elucidate the crosstalk between the microenvironments and cells/tissues. Understanding hydrogel properties is also important to determine its biomedical applicants. Special attention has been given to the development of dynamic hydrogels, which properties can be modulated in response to external stimuli^3^,^4^,^5^. Regulation of temporal and spatial properties has allowed for the manipulation of synthetic hydrogel properties^6^,^7^. The regulation of protein conformation is an alternative strategy for dynamic property control within protein-based hydrogels^8^,^9^,^10^. Protein conformational alternations may occur naturally in the human body, some of them are pathological hallmarks for serious diseases^11^. An example is the formation of amyloid plaque, associated with neurodegenerative disorders, such as Parkinson’s and Alzheimer’s^12^. It is critical to understand how cells and/or tissues behave in response to protein conformational changes via *in vitro* models mimicking human physiological conditions. The use of protein-based hydrogels would be an advantageous biomimetic solution. Thus far, there are no reports on such uses; also most *in vitro* amyloid models available are limited to the β-amyloid (Aβ) peptides.

Silk fibroin (SF) made from *Bombyx mori* silkworm have been extensively studied as a versatile biomaterial for tissue engineering as it possess superior mechanical properties and biodegradable^13^,^14^. Usually, SF hydrogels are formed through the transition from random coil structure to β-sheet, which may happen by adding solvents, decreasing pH or increasing the temperature/ion concentration in the aqueous silk solution^15^,^16^,^17^,^18^. Most recently, a horseradish peroxidase (HRP) mediated crosslinking approach has shown to be promising for preparation of injectable hydrogels. The polymers that compose the injectable hydrogels have tyrosine groups exposed under physiological conditions^19^. SF contains approximately 5% tyrosine groups which can be used for crosslinking via HRP catalyzed reaction. Anderson reported firstly the formation of di- and trityrosine in silk fibroin by using this method and found that rigid SF hydrogels can be formed in 0.1 M sodium bicarbonate solution^20^,^21^,^22^,^23^. Following, Aeschbach *et al*. studied the formation of dityrosine in SF and other 14 proteins via oxidation of tyrosine residues by HRP and hydrogen peroxide catalysis in basic pH^22^. However, these studies neither showed detailed physicochemical properties nor the biomedical application of the formed SF hydrogels. Our group conducted the first systematic study on the SF hydrogels via HRP catalysis in physiological conditions^24^,^25^. In our issued patent (submitted in 2011), the physicochemical properties of SF hydrogels prepared from SF solution of varied concentrations were characterized, and initial cell encapsulation results (up to 7 days) were also presented^25^.

Here we describe that the SF hydrogel system, which was developed in our group by HRP catalyzed crosslinking, can undergo spontaneous conformational change (after 7 days) under physiological conditions. The freshly fabricated hydrogels presented dominant random coil conformation and were transparent and highly elastic. Furthermore, the hydrogels displayed dual ionic strength and pH responses. We found that these biomimetic hydrogels acquire a β-sheet conformation after 10 days under physiological conditions *in vitro*. We further explored the *in vivo* conformational alteration behaviour of the SF hydrogels and their *in vivo* biocompatibility by subcutaneous implantation in rats for 14 days. These hydrogels also became β-sheet *in vivo*. Thus, we would like to know the cell behaviour in the random coil SF hydrogels and how the cells respond to the protein conformation change. In the first step, ATDC-5 cells were used and the SF hydrogels allowed for *in vitro* cell encapsulation. We considered these hydrogels may be used as a 3 dimensional (3D) scaffold for cancer research^26^. Next we investigated the effect of conformational changes in tumor formation and growth. Here, a chick chorioallantoic membrane (CAM) model was used to assess the effect of SF conformational changes in encapsulated HeLa cells within the hydrogel.

## Results

### Random Coil Conformation and Dual Stimuli Response of the SF hydrogels

**Figure 1:**
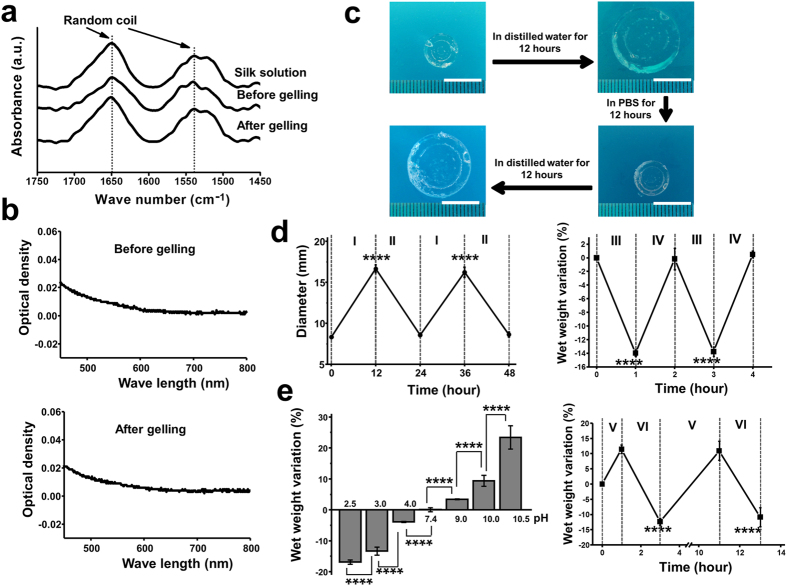
SF hydrogel structural analysis, optical absorbance and response to stimuli. (**a**) ATR-FTIR spectra for the aqueous SF solution, SF/HRP/H_2_O_2_ mixture before gelling, and after SF hydrogel formation. (**b**) Visible light absorbance for the SF/HRP/H_2_O_2_ before gelling and for formed SF hydrogel, respectively. (**c**) Shape memory property for the SF hydrogels tested by alternatively immersing the hydrogel disc in distilled water and PBS (Scale bar: 1 cm). (**d**) SF hydrogel response to changing ionic strengths. Left: Diameter changes during the alternate immersion in distilled water (I) and PBS (*n* = 5). Right: Wet weight variation during alternative immersion in 2.0 M (III) and 0.154 M sodium chloride solutions (both of pH 7.4) (*n* = 5). (**e**) SF hydrogel pH response. Left: Wet weight variation after immersion in solutions of different pH values for 2 hours, respectively (*n* = 5). Right: Wet weight variation during the alternate immersion in basic (pH 10.5, V) and acid (pH 3.0, VI) sodium chloride solutions (*n* = 6). ****P < 0.0001.

SF hydrogel conformation was analysed by attenuated total reflectance Fourier transform infrared spectroscopy (ATR-FTIR, Fig. 1a). The main absorbance peaks for the silk solution, the silk/HRP/H_2_O_2_ mixture before gelling and the hydrogel were all at 1650 cm^−1^ and 1538 cm^−1^. These peaks were assigned to the random coil conformation as reported previously^27^,^28^,^29^. Unlike the opaque β-sheet SF hydrogels^17^, our hydrogels presented an optical absorbance between 0 to 0.2 before or after gelling (Fig. 1b). The low absorbance of visible light is an indicator of amorphous conformation^17^.

Strikingly, these hydrogels demonstrated shape memory as they reversibly swelled and shrank when alternatively immersed in distilled water and PBS, respectively (Fig. 1c). We further conducted quantitative studies by measuring the diameter changes of the hydrogels when alternatively immersion the hydrogels in distilled water and PBS. The hydrogel diameter was recovered to almost the same value during different immersion cycles (Fig. 1d, left). The diameter of the hydrogels increased around 100% after immersion in distilled water for 12 hours. Additionally, we studied the swelling behaviour of the SF hydrogels in sodium chloride solutions of physiological and higher than physiological concentrations. It was found that the wet weight of these hydrogels responded to sodium chloride solutions of varied concentration (Fig. 1d, right). There was around 14% decrease in the wet weight of the hydrogels when immersion in 2 M sodium chloride solution for 1 hour. The diameter and wet weight changes in different solutions indicated these hydrogels were of ionic strength response. We further found that these hydrogels were pH responsive. They swelled in basic conditions and shrank in acid solutions (Fig. 1e, left). When decreasing the pH from 7.4 to 2.5, the wet weight of the hydrogels reduced around 17% in 2 hours. While enhancing the pH from 7.4 to 10.5, about 22% increase in the wet weight of the hydrogels was observed. Furthermore, the wet weight of the hydrogel can be reversed when alternatively immersed in solutions of pH 10.5 and pH 3.0 (Fig. 1e, right). These random coil hydrogels display a fast degradation profile when soaked in a solution of protease XIV (Figure S1). After degradation for 4 hours, the hydrogels lost around 50% of their wet weights and completely degraded after 12 hours.

### Short Gelling Time and High Elasticity of the SF Hydrogels

**Figure 2:**
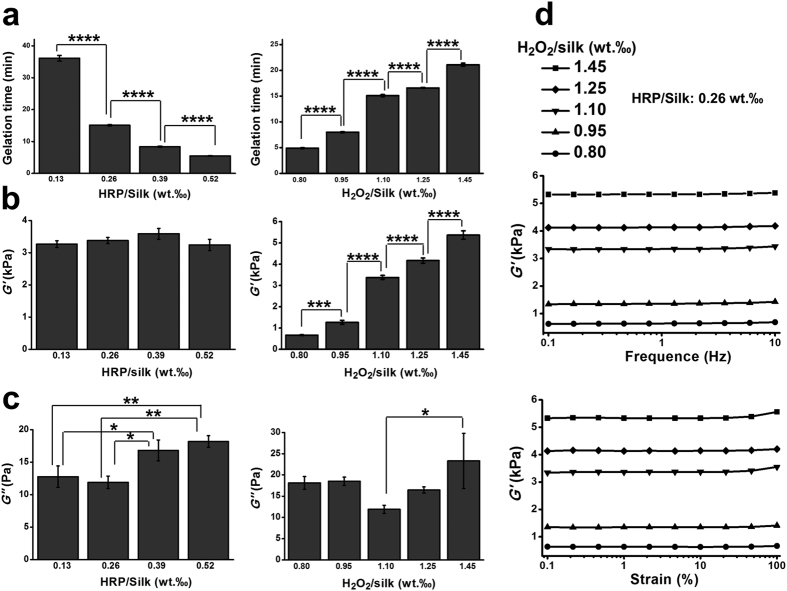
Gelling time and SF hydrogel mechanical properties. (**a**) The HRP and H_2_O_2_ influence on the hydrogel gelling time. (**b**,**c**) the HRP and H_2_O_2_ effects on the storage contents and the hydrogel’s loss modulus, respectively. In (**a**–**c**), Left: H_2_O_2_/Silk was fixed at 1.10‰ (wt/wt); Right: HRP/Silk was fixed at 0.26‰ (wt/wt). (**d**) hydrogel representative frequency and strain sweeping. (**a**–**c**: *n* = 5). *P < 0.05, **P < 0.01, ****P < 0.0001.

The influences HRP and H_2_O_2_ contents on the gelling time and mechanical properties of the hydrogels were studied. The SF hydrogel gelling time significantly decreased (P < 0.0001, comparing any two groups among the four groups) as the HRP content increased (Fig. 2a, left). As increasing the HRP content from 0.13‰ to 0.52‰, the gelling time decreased from around 36 minutes to 5.5 minutes (P < 0.0001, comparing any two groups among the four groups). An opposite trend was observed in the gelling time (P < 0.0001, comparing any two groups among the five groups) when increasing the H_2_O_2_ content (Fig. 2a, right). When improving the H_2_O_2_ content from 0.8‰ to 1.45‰, the gelling time improved from less than 5 minutes to 21 minute. We study the SF hydrogels mechanical properties by rheometer^30^. The hydrogel storage modulus (*G*’) did not show significant differences when changing the HRP content (Fig. 2b left). In comparison, the *G*’ greatly improved as the H_2_O_2_ content increased (P < 0.0001, comparing any two groups among the five groups) which can be shaped within a range of several hundred Pa to 5 kPa (Fig. 2b, right). The SF hydrogel loss moduli (*G”*) were in a range of 10 to 21 Pa (Fig. 2c). A higher *G*’*/G*” value indicated superior elasticity of these hydrogels. In both the frequency and strain sweeping studies, a steady representative *G*’ profile was observed in each formulation of the hydrogels (Fig. 2d), further confirming the high stability and elasticity of these hydrogels.

### *In Vitro* and *In Vivo* Conformation Alternation of the SF Hydrogels

**Figure 3:**
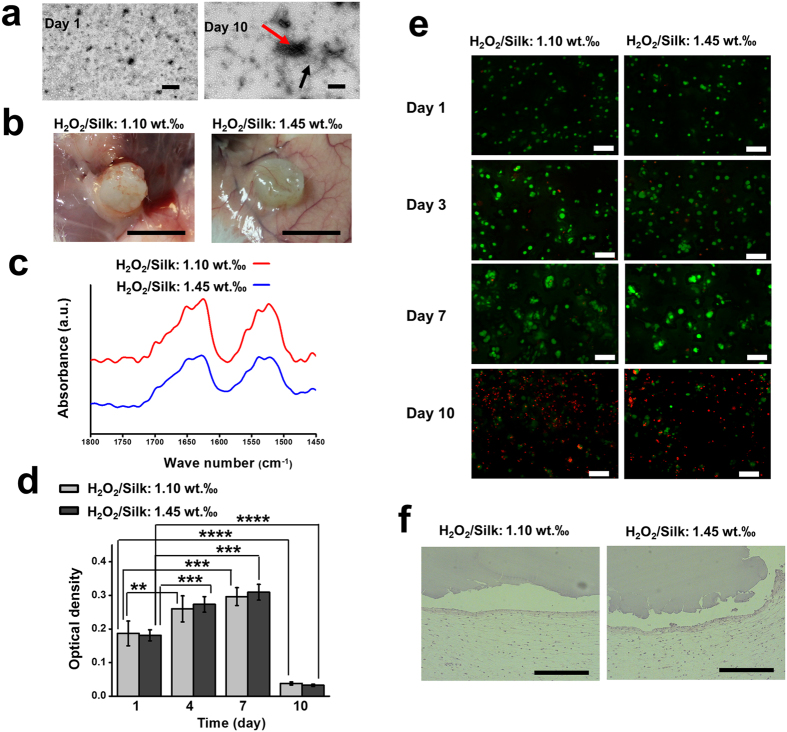
*In vitro* conformation evaluation and cell encapsulation studies and *in vivo* subcutaneous implantation for the SF hydrogels. (**a**) SF hydrogel transmission electron micrographs after incubation in PBS at 37 °C for 1 (left) and 10 days (right). The black arrows indicate nanofibrils and the red arrows indicate nanofibrils aggregates (Scale bar:200 nm). (**b**) Macroscopic images of explants retrieved after 2 weeks of subcutaneous implantation (scale bar: 5 mm). (**c**) SF hydrogel ATR-FTIR spectra after 2 weeks of implantation. SF solution: 16 wt. %; Silk/HRP: 0.26‰ (wt/wt). (**d**) Cell viability after encapsulation analyzed by MTS assay (*n* = 9). (**e**) Live/dead staining for the ATDC-5 cells whithin the SF hydrogels from 1 to 10 days (scale bar: 100 μm). (**f**) H&E staining of the explants (scale bar: 400 μm). **P < 0.01, ***P < 0.001, ****P < 0.0001.

When immersion of the SF hydrogels in PBS at 37 °C, we found that these hydrogels became opaque after 10 days. We further studied the microstructure differences between the transparent and opaque SF hydrogels by Transmission electron microscopy (TEM). The TEM images revealed the presence of a small amount of non-bound SF nanofibrils in the hydrogels at day 1 (Fig. 3a, left), implying superior amorphous conformation in these hydrogels. However, a substantial increase in the dimensions of the nanofibrils and their aggregates, typically found on a β-sheet transition^31^, were observed in the hydrogels after 10 days (Fig. 3a, right).

We also investigated the *in vivo* conformation transition behaviour of the SF hydrogels by implantation the hydrogels subcutaneously in rat for 14 days. Overtime the subcutaneous implanted SF hydrogels changed from elastic and transparent to stiff and opaque appearance (Fig. 3b). ATR-FTIR spectra of the SF hydrogels explants presented strong peaks located at 1627 cm^−1^ and 1522 cm^−1^ (Fig. 3c), which were assigned to the β-sheet conformation^29^. Therefore, these results indicate that the conformation of the SF hydrogels can spontaneously change to β-sheet over time both *in vitro* and *in vivo*. Additionally, the subcutaneous implantation results also showed that there was no acute inflammation promoted by the SF hydrogels, indicated their desirable *in vivo* biocompatibility (Fig. 3f).

### *In Vitro* Encapsulation of ATDC-5 Cells

Due to a short gelling time, the hydrogels developed here were used for cell encapsulation. The MTS evaluation shows that the metabolic activity of the encapsulated ATDC-5 cells improved from day 1 to day 4 (P < 0.01 and P < 0.001 for the H_2_O_2_/silk 1.10‰ and H_2_O_2_/silk 1.45‰ groups, respectively), and was maintained until day 7 (Fig. 3d). However, cell viability decreased dramatically at day 10 compared to day 1 (P < 0.0001 for both H_2_O_2_/silk 1.10‰ and H_2_O_2_/silk 1.45‰ groups). From the Live/Dead assay staining, it was found that most of the incorporated cells were alive until to 7 days in all formulations (Fig. 3e). However, large amounts of dead cells were found at day 10 in both formulations. As shown in Figure S2a,b, the cell-laden SF hydrogels were stable and maintained its transparency until day 6. However, the hydrogels became opaque at 10 (Figure S2c). The hydrogel SEM images show a porous structure at day 6 (Figure S3a) and a dense morphology at day 10 (Figure S3b). These results present unequivocal evidence of conversion to dominant β-sheet conformation in the SF hydrogels. Our hypothesis was that conformational changes induced apoptosis of encapsulated cells.

### *In Vitro* Encapsulation of HeLa cells

**Figure 4:**
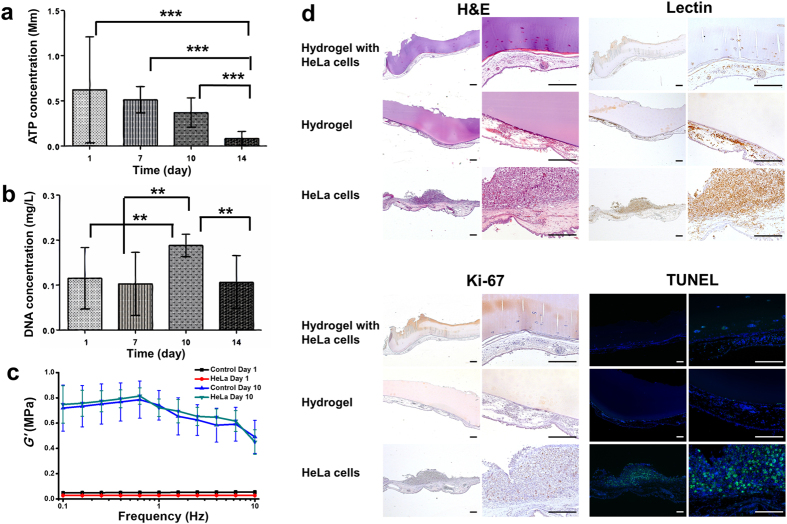
HeLa cell encapsulation and SF hydrogel anti-angiogenic effect. (**a**) Cell proliferation and viability measured by (**a**) ATP assay and (**b**) DNA quantification, respectively, for SF hydrogel with encapsulated HeLa cells after 1, 7, 10 and 14 days of culture (*n* = 9). (**c**) storage modulus for the cancerous cell laden-hydrogels, tested by dynamic mechanical analysis (n = 3). Control Day 1 and Control Day 10: hydrogels without cells encapsulated freshly prepared and 10 days post-preparation; HeLa Day 1 and HeLa Day 10: hydrogels encapsulated with HeLa cells for one day and ten days, respectively. (**d**) H&E staining, SNA-lectin and Ki-67 immunohistochemical analysis and fluoresence TUNEL assay of the excised section from the CAM study (scale bar: 200 μm). **P < 0.01, ***P < 0.001.

Subsequently, we intended to explore the application of the conformational changes of the SF hydrogels in angiogenic suppression and anti-tumor studies. At first, HeLa cells were loaded into the hydrogels. The ATP measurements demonstrated that the number of viable cells decreased with time in culture (Fig. 4a). A significant decrease of metabolically active cells was observed at day 14 compared to other days (P < 0.001). From DNA quantification analysis (Fig. 4b), it was observed a significant increase in cell proliferation after 10 days. However, no significant differences were found between day 1 and day 7. A significant decrease in the DNA content was verified from day 10 to day 14 (P < 0.01), which is consistent with the results obtained from ATP measurements (Fig. 4a). The cell-loaded hydrogels at first instance were transparent; 7 days later the gels had an irregular opacity throughout the disc. Furthermore, the dynamic mechanical analysis shown that the storage modulus of the cell-laden hydrogels enhanced substantially after 10 days as compared to day 1 (Fig. 4c). The loss factor of the hydrogels increased dramatically from day 1 to day 10, indicating less elasticity of the hydrogels after acquiring β-sheet conformation (Figure S4).

### Suppression Angiogenesis and Anti-Tumor Formation of the SF Hydrogels

In the next step, the HeLa cell-laden SF hydrogels were implanted in a CAM model. CAM is extensively used as a model for tumor biology^32^. Here we investigate the potential of SF hydrogels as a biomimetic 3D model for inducing cancer cells apoptosis mediated by hydrogel conformational transition. H&E staining (Fig. 4d, upper left) shows that only a few cell clusters were detected inside the cell-laden hydrogels. Additionally, the SF hydrogels did not allow endothelial cells infiltration in the cell free hydrogel. As expected, the formation of a solid tumor was observed on the CAM of embryos implanted with HeLa cells. Immunohistochemical analysis was performed against SNA-lectin (for chick endothelial cells) and Ki67 (for human proliferating cells). Only small agglomerates containing cells were observed in the cellular hydrogels, which were negative for the SNA-lectin staining (Fig. 4d, upper right). No endothelial cells were found inside the SF hydrogels, indicating that the hydrogel does not allow cells infiltration and vessel formation, which is critical for tumor development. As expected, a massive positive staining for endothelial cells was found in the embryos implanted with HeLa cells, in the region where the solid tumor formed. In this system, a small number of cells forming agglomerates within the hydrogels were positive for human proliferative cells (Fig. 4d, lower left). No Ki67 staining was detected in the cell free hydrogel. Moreover, a considerable number of Ki67-positive cells were observed in the solid tumor region. Cell apoptosis was detected by using the TUNEL assay (Fig. 4d, lower right). Some apoptotic cells were detected within the cell-loaded hydrogels, whereas no positive staining was observed in the cell free hydrogel explants. A high number of apoptotic cells were detected in the formed solid tumor. Although the positive staining pattern was distinct from the one observed in the positive control (Figure S5b), this type of positive staining pattern has already been described^33^.

## Discussion

Injectable hydrogels prepared by HRP mediated crosslinking have been attracting great interest in tissue engineering and regenerative medicine^19^. Varied biopolymer hydrogels have been developed by this approach, such as dextran, alginate, gelatin, and chitosan^34^,^35^,^36^,^37^. In the present study, we developed a new class of SF hydrogels by HRP medicated crosslinking. These hydrogels presented completely distinct properties compared to the SF hydrogels of β-sheet conformation presented in the literature^16^,^17^,^38^. Table 1 compared SF hydrogels prepared by different methods. It was found that almost all the previous developed SF hydrogels were of β-sheet conformation, except the electrically induced SF hydrogel and a recent study from Partlow *et al*.^39^. However, the electrically induced hydrogels were not suitable for cell encapsulation, since the direct current used for gelation and the unstable mechanical properties of the hydrogels^27^. In an important study from Partlow *et al*., highly elastic SF hydrogels of random coil conformation were prepared by using HRP mediated crosslinking. In that study, hydrogels were prepared from 1–6% silk water solution and studied after 1 hour gelling time. The SF hydrogels developed in current study were also of random coil conformation. Compared to the study from Partlow *et al*.^39^, our SF hydrogels were prepared from a higher concentration silk solution (16%) and in a physiological conditions. The usage of a higher silk solution led to a shorter gelation time (between 4–40 minutes) of the resulted hydrogels. In our initial trial study, we found that SF hydrogels prepared with 10% and 12% aqueous SF solution showed longer gelation time than the ones prepared from 16% SF solution (data not shown). Thus, the shorter gelation time in current study compared to previous reported study may also because more tyrosine groups were available in the system for crosslinking^39^. Additionally, a short gelation time and physiological conditions are desired in development of injectable hydrogels for tissue substitute, cell encapsulation and drug delivery.

Another interesting characteristic of our SF hydrogels is the dual-stimuli response, namely ionic strength and pH stimuli response. These properties are unprecedented for SF hydrogels of β-sheet conformation and are based on their dominant amorphous conformation. Partlow *et al*. also mentioned the ionic strength response of SF hydrogel in their study, while the response extent in their study is much less than the one in our study^39^. The transparent feature and stimuli response of the SF hydrogels may open a new window for applying SF as biosensors via microfabrication technique.

Mechanical properties of biomaterials are critical for their applications in tissue regeneration. Normally, physical crosslinked SF hydrogels are stiff but brittle, lacking adequate elasticity required for many tissue repair, such as cartilage, skin and adipose tissue. In this study, we showed that the SF hydrogels were highly resilient and their mechanical properties can be easily harnessed in a wide range only by changing the contents of hydrogen peroxide. The mechanical properties of the SF hydrogels also somehow reflected the crosslinking degree of the hydrogels networks. Higher content of hydrogen oxide in the system can induce higher amount of oxidized tyrosine groups which were involved in the crosslinking process and thus resulted in the enhanced crosslinking degree. The enhanced crosslinking degree in the SF network would lead to improved mechanical properties of the hydrogels (as showed in Fig. 2b, right). Previously, Partlow *et al*. adjusted the modulus of SF hydrogels ranged from around 1 to 10 kPa by varying the SF concentration and degumming time (molecular weight)^39^. They also presented that the SF hydrogels undergone 30 or 60 minutes degumming treatment showed similar storage modulus ranged from about 1–5 kPa. In current study, we can controlled the modulus from several hundred Pa to around 6 kPa. In both studies, the SF hydrogels maintained their superior elasticity under dynamic strain and frequency sweeps. The underlying reason for these features in the SF hydrogels are their random coil conformations.

The degradation behavior of biomaterials is an essential properties for their application in tissue regeneration. Our developed SF hydrogels, which is of main random coil conformation, showed fast weight loss profile in the *in vitro* enzymatical degradation (Figure S1). But they were quite stable in the first 6 days of the *in vitro* cell encapsulation study. During this time period, they were still of main random coil conformation (Figure S2). Furthermore, the subcutaneous implantation results showed that these hydrogels did not collapse or present obvious degradation (Fig. 3b). This result indicated that these hydrogels in their random coil status were resistant to *in vivo* degradation before the β-sheet transition. Combining the *in vitro* and *in vivo* observations, our hydrogels possess desirable stability as a novel biomaterials for biomedical application.

One more advantage of the SF prepared in this study is that they allowed cell encapsulation and presented desired *in vivo* biocompatibility. SF hydrogels of β-sheet conformation are usually not compatible for cell encapsulation as the harsh preparation procedure, excluding the sonication induced SF hydrogels^38^. As the HRP mediated crosslinking is performed in physiological condition and the SF is in random coil conformation, cells can be easily encapsulated. The SF hydrogels are cytocompatible as they can support the cell survival up to 7 days. The superior cytocompatibility and *in vivo* compatibility confirmed that SF is a promising biomaterials for tissue engineering and regenerative medicine.

Especially, we discovered that the random coil SF hydrogels would eventually turned into β-sheet conformation *in vitro* and *in vivo* as confirmed by FTIR-ATR and TEM assays. Amorphous aqueous SF solution would inevitably acquire a β-sheet conformation overtime^16^, while the solution itself cannot provide a stable support for studying the cell behavior. Previous studies had applied protein hydrogels of specific conformation for cell encapsulation^40^,^41^, while the influence of conformation change on cell fate during cell culture has never been reported. In this study, for the first time, we showed that the enzymatically crosslinked SF hydrogels can be used as a model to investigate the influence of protein conformational on cell fate. Our results demonstrated that the hydrogel conformation change from amorphous to β-sheet resulted in apoptosis of ATDC-5 cells (Fig. 3d).

We further attempted to apply the conformation change property of these hydrogels for suppression tumor formation study. Cell apoptosis is a vital process in tumor cell dynamics and response to anti-cancer treatments. Therefore evaluation of apoptosis is of interest for biological evaluation of tumor kinetics and prognosis to treatment. On the other hand, angiogenesis is an essential step for tumor growth and metastasis and a valuable strategy for cancer therapy would be the inhibition of tumor angiogenesis^42^. The results obtained in the CAM assay indicate that the SF hydrogels do not allow tumor formation after the hydrogels acquire the β-sheet conformational, which prohibited the invasion of endothelial cells and thus limited blood supply for tumor growth. The small number of cells found in the cell-loaded SF hydrogels can be attributed to the mechanical environment changes from the conformational transition process (Fig. 4c). Our observations showed that the modulus of the cancer cells-laden hydrogels increased dramatically after β-sheet transition. The substantial change of the mechanical environment in the hdyrogels led to the apoptosis of the cancer cells, prohibited angiogenesis in the hydrogels and thus suppressed tumor formation in the hydrogels.

## Conclusion

In this study, we developed SF hydrogels of dominant random coil conformation and dual-stimuli responsive properties via enzymatically crosslinking approach. These SF hydrogels are injectable, highly resilient and presented widely tunable mechanical properties. They are able to be used as cell encapsulation system and displayed desired *in vivo* biocompatibility. Importantly, these hydrogels would spontaneously acquire conformation change which allowed these hydrogels to serve as a biomimetic platform for modulation of encapsulated cell fate and suppression of cancer formation. Therefore, this study does not only highlights new insights for the fundamental study of hydrogel-based 3D models in cancer research, but also provides a new biomaterial candidate for diverse applications, such as spatiotemporal drug delivery, tissue regeneration and regenerative medicine.

## Methods

### Materials and reagents

Cocoons of *Bombyx mori* were provided by the Portuguese Association of Parents and Friends of Mentally Disabled Citizens (Portugal). Horseradish peroxidase (HRP, type VI, 260 U/mg) was purchased from Sigma-Aldrich (St. Louis, MO, USA). All the other reagents or materials were supplied by Sigma-Aldrich unless otherwise stated.

### Preparation of silk fibroin solution and SF hydrogels

The purified silk fibroin (SF) was dissolved in 9.3 M lithium bromide, followed by dialysis against distilled water for 48 hours^43^. Then the SF solution was dialyzed in phosphate buffered saline solution (PBS) for 12 hours before concentration it using a 20 wt.% poly(ethylene glycol) solution. HRP solution (0.84 mg/mL) and hydrogen peroxide solution (H_2_O_2_, 0.36 wt.%) were both prepared in PBS. SF solution of 16 wt.% concentration was used for the hydrogel preparation. SF hydrogels were prepared by mixing 1 mL of SF solution with varied amount of HRP and H_2_O_2_ solutions in a centrifuge tube in a water bath at 37 °C. The gelling time was determined by inverting the vial (n = 5). SF hydrogel discs were also prepared by adding of 200 μL of the mixture solutions in a polypropylene mold at 37 °C. The SF hydrogels were denoted as Silk-16.

### Structural characterization of the SF hydrogels

HRP/Silk and H_2_O_2_/Silk were fixed at 0.26‰ and 1.1‰ (wt/wt) respectively for sample preparation followed by structural characterization. The SF solution, a mixture of SF/HRP/H_2_O_2_, and the formed SF hydrogels were analyzed by attenuated total reflectance (ATR) in a Fourier transform infrared spectroscopy (FTIR) (IRPrestige-21, Shimadzu, Kyoto, Japan). The SF optical absorbance was further recorded in a microplate reader (Synergy HT, BioTek Instruments, Winooski, VT, USA). The optical absorbance of 50 μL mixture was red before and after gelling from 450 to 800 nm.

### Degradation profile of the SF hydrogels

Three formulations of the hydrogel discs were used for degradation study: 1/0.26‰/1.1‰, 1/0.52‰/1.1‰, and 1/0.26‰/1.45‰ (Silk/HRP/H_2_O_2_). The wet weight of each specimen was recorded before the addition of 5 mL protease XIV solution (0.005 U/mL). Then, the degradation was conducted in a water bath at 37 °C. The wet weight of each specimen was measured after 1, 2, 4, 6, and 12 hours. The ratio of weight loss was determined according to Equation (1):
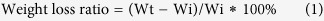
In Equation (1), Wi indicates the initial hydrogel wet weight, and Wt is the wet weight measured at different time points (n = 5).

### Stimuli responsiveness of the SF hydrogels

Hydrogels in the formulation of 1/0.26‰/1.45‰ (Silk/HRP/H_2_O_2_) were used for the stimulus response studies. All the procedures were performed at 37 °C.

#### Ionic strength response

In the first immersion sequence, each hydrogel disc was alternately immersed in distilled water and PBS every 12 hours. Before every change of the liquid, the hydrogel diameter was measured. For the second immersion sequence, the prepared hydrogels were alternately immersed in a 100 mL 2 M sodium chloride solution (pH 7.4) and a 100 mL 0.154 M sodium chloride solution (pH 7.4) every hour. Before changing the solutions, the hydrogel’s wet weight was recorded. The wet weight variation ratio was calculated as following in Equation (2)
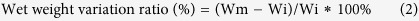
In Equation (2), Wi means initial hydrogel weight after overnight immersion in 0.154 M sodium chloride solution, and Wm refers to the wet weight measured during the alternated immersion in 2 M and 0.154 M sodium chloride solutions (n = 5).

#### pH response

In the first part, the hydrogels were immersed in sodium chloride solutions of different pH values: 2.5, 3.0, 4.0, 7.4, 9.0, 10.0 and 10.5. After 2 hours, the hydrogel’s wet weights were recorded (n = 5). In the second part, the hydrogel discs (n = 6) were alternately immersed in basic (pH 10.5) and acid (pH 3.0) sodium chloride solutions, after measuring the initial wet weight. Before each solution change, the weights of the samples (wet) were registered. The wet weight variation ratio was calculated as indicated in Equation (1).

### Mechanical property determination

The hydrogel storage and loss of moduli were evaluated by using an oscillatory model in a rheometer (MCR 300, Anton Paar, Graz, Austria). For each measurement, 1 mL SF solution was mixed with varied amount HRP and H_2_O_2_, and then 1 mL of the mixture was transferred into the rheometer for evaluation. For the modules measurement, the time sweep was first performed under constant strain (0.1%) and frequency (0.5 Hz) until the gel formed. After the storage modulus plateau was reached, the frequency sweep (from 0.1–100 Hz) was conducted for 5 minutes with the strain fixed at 0.1%. The strain sweep (0.1–100%) was done following the frequency sweep and measured for another 5 minutes under constant frequency at 1 Hz. All measurements were conducted at 37 °C (n = 5).

### *In vitro* β-sheet conformation transition

The *in vitro* conformation transition on SF hydrogels was also analyzed by Transmission Electron Microscopy (TEM) (JEOL JEM-1400, Tokyo, Japan). The hydrogels were incubated in PBS at 37 °C for 1 and 10 days, respectively. The hydrogel formulation used was 1/0.26‰/1.45‰ (Silk/HRP/H_2_O_2_).

### *In vivo* implantation

The maintenance and use of animals in current study were according to European Council Directives on Animal Care (Directive 2010/63/EU). The animal experiments and protocols used in this study were approved by the Ethics Committee of University of Minho. Two formulations were used for *in vivo* implantation: 1/0.26‰/1.1‰ and 1/0.26‰/1.45‰ (Silk/HRP/H_2_O_2_). A SF solution of 16 wt.% was sterilized by UV radiation for 15 minutes in a sterile cabinet. In each mouse (Charles River, Senneville, Quebec, Canada), 4 skin incisions were made on the back following standard procedure. Afterwards, 4 hydrogel discs were implanted subcutaneously into the respective pockets and the skin was sutured. For each formulation, 8 pieces of hydrogel discs were implanted. The mice were euthanized and the implants were retrieved (2 weeks post-surgery). The explants (n = 5) were fixed with a 10 vol.% formalin solution, followed by dehydration. Then the samples were embedded in paraffin and slides were prepared into 5 μm-thick sections using a microtome. The sections were stained with Hematoxylin and Eosin (H&E). Additionally, the retrieved SF hydrogels (n = 3) were analyzed by ATR-FTIR.

### Cell encapsulation and cytotoxicity

ATDC-5 cell pellet containing 1 × 10^6^ cells was obtained after removing the supernatant. The SF solution (1 mL) was mixed with the HRP and H_2_O_2_ solutions and the mixture was warmed in the water bath (37 °C) for 6 minutes. Two formulations were used: 1/0.26‰/1.1‰ and 1/0.26‰/1.45‰ (Silk/HRP/H_2_O_2_). The mixture was mixed with the cell pellet, then 50 μL of the cell suspension was transferred into coverslips placed in a 24-well suspension cell culture plate. The plate was then incubated for 10 minutes to allow gelling. Following, basal MEM alpha medium was added to each well, and the medium was changed every two days. Cell viability was evaluated by MTS (Promega, Fitchburg, WI, USA), at day 1, 4, 7, and 10 (n = 9). The viability of encapsulated cells was confirmed by Calcein AM and propidium iodide (Molecular Probes; Life Technologies, Carlsbad, CA, USA) staining. The cell-laden hydrogels were frozen in liquid nitrogen and then lyophilized, after culturing for 6 and 10 days, respectively. The morphology of the hydrogels was observed by scanning electron microscopy (SEM, Nova NanoSEM 200, FEI, Hillsboro, OR, USA).

### Culture of HeLa cells and preparation of cell-loaded hydrogels

HeLa cell line was generously donated by Dr. Elsa Logarinho from the Institute for Molecular and Cell Biology (IBMC), Porto, Portugal. The experiments and protocols in this study related with HeLa cell line were approved by the Ethics Committee of University of Minho. An aqueous SF solution (16 wt.%) was used for cancer cell encapsulation. Cancer cell encapsulation was performed using the same protocol for ATDC-5 cells encapsulation. The hydrogel formulation used was 1/0.52‰/1.45‰ (Silk/HRP/H_2_O_2_).

### *In vitro* evaluation of viability and proliferation of HeLa cells in the hydrogels

Cell viability was assessed after 1, 7, 10 and 14 days of culture, using the CellTiter-Glo Luminescent Cell Viability Assay (Promega, Madison, WI, USA) (n = 9). Samples without cells were used as control. Cell proliferation was quantified using the Quant-iT Pico-Green dsDNA Assay Kit (Life Technologies, Carlsbad, CA, USA), according to the manufacturer’s instructions (n = 9). The fluorescence intensity was measured in a microplate reader. Hydrogels without cells were used as the control group.

### Dynamic mechanical analysis (DMA) of Hela cells-laden hydrogels

The mechanical properties of the cell-loaded SF hydrogels were determined using a Tritec 8000B dynamic mechanical analyzer (DMA; Triton Technology, UK) in the compressive mode. Hydrogels (cylindrical shape of 6 mm diameter and 2 mm thickness) encapsulated with HeLa cells (1 × 10^5^ cells per 100 μL of hydrogel) were tested after 1 and 10 days of culture (n = 3).

### Chorioallantoic membrane (CAM) assay

CAM assay was performed according to the procedure described by Silva-Correia *et al*.^33^ in a laminar-flow hood to minimize contamination. Briefly, white fertilized chicken eggs (*n* = 90–120) were incubated at 37 °C for 3 days. A small hole was created in the pointed end of the egg and a circular window was made into the egg-shell. Sterile hydrogel discs (minimum of 18 per condition in each assay) after culturing for 4 days were then implanted in the CAM at day 10 of embryonic development. Three experimental conditions were implanted: Silk-16 and Silk-16+HeLa cells hydrogel discs and HeLa cells (2 × 10^6 ^cells/egg as control for tumor formation). The eggs were returned to the incubator at 37 °C until day 17 of embryonic development. The CAM and underlying embryos were fixed *in ovo* by using paraformaldehyde solution, followed by incubation at −80 °C for 10 minutes. The implanted discs (or tumor in control group) and the immediately adjacent CAM portions were cut and transferred to 4% paraformaldehyde solution. The excised membranes were embedded in paraffin and serially sectioned for immunohistochemical analysis. Three independent CAM assays were performed.

### Histological and immunohistochemical staining

#### H&E staining

The CAM histological sagittal sections (4 μm-thick) were stained with H&E and observed under transmitted microscopy using an Axio Imager Z1m light microscope (Zeiss, Oberkochen, Germany).

#### SNA-lectin

CAM sections containing the hydrogel were subjected to immunohistochemical analysis for biotinylated *Sambucus Nigra* (Elderberry) bark lectin (SNA, EBL) according to the streptavidin-biotin peroxidase complex system. SNA-lectin specifically binds to chick endothelial cells. The immune reaction was visualized by using the chromogen 3,3′-Diamonobenzidine (DAB; Thermo Fisher Scientific, Waltham, MA, USA) and after counterstaining with Gill-2 haematoxylin (Merck, Darmstadt, Germany).

#### Ki67

CAM sections were used for immunohistochemical analysis for Ki67 (SP6; Gennova Scientific, Sevilla, Spain) according to Avidin-biotin-peroxidase principle. Ki67 is a nuclear protein providing a measure of cells that have entered the cell cycle. The immune reaction was visualized with 3,3-Diamonobenzidine as a chromogen. All sections were counterstained with Gill-2 haematoxylin.

#### TUNEL assay

The *In situ* Cell Death Detection Kit, Fluorescein (Roche, Basel, Switzerland) was used in CAM sections to detect apoptotic cells, based on Terminal deoxynucleotidyl transferase dUTP nick end labeling (TUNEL) reaction and according to manufacturer’s instructions. A counterstaining with DAPI was also performed. Apoptotic cells were observed and imaged as described above.

### Statistical analysis

The data is presented by mean ± standard deviation (SD). The results from the ionic strength response, pH response, gelling time and rheological analysis were analyzed first by Kolmogorov-Smirnov test to confirm the normal distribution, following by one-way analysis of variance (ANOVA) and a Bonferroni post-hoc analysis. The MTS result was analyzed by using non-parametric Mann-Whitney test. For the ATP and DNA quantification assays, the differences between the experimental results were analyzed using a Kruskal-Wallis test followed by Dunn’s multiple comparison test. The significance level was *P < 0.05, **P < 0.01, ***P < 0.001, ****P < 0.0001.

## Additional Information

**How to cite this article**: Yan, L.P. *et al*. Tumor Growth Suppression Induced by Biomimetic Silk Fibroin Hydrogels. *Sci. Rep.* **6**, 31037; doi: 10.1038/srep31037 (2016).

## Supplementary Material

Supplementary Information

## Figures and Tables

**Table 1:** Comparison of SF hydrogels.

Ref.	Method	Shortest gelation time	Modulus (kPa)	Main conformation	SF concentration
16	Storing SF solution at 4 °C	~3 days	—	β-sheet	2 wt.%
16	Addition of glycerol in SF solution	~2 days	—	β-sheet	2 wt.%
44	Addition of citric acid in SF solution and storing at 50 °C	Overnight	—	β-sheet	2 wt.%
15	Increasing SF concentration or temperature, decreasing pH, addition of ions, or polyethylene glycol	＜1 day	~200–6000 (C)	β-sheet	4–20 wt.%
17	Freezing the SF solution with organic solvents at −20 °C	> 6 hours	~3–50 (C)	β-sheet	6 wt.%
37 a	Sonication treatment on SF solution	>0.5 hour	369–1712 (C)	β-sheet	4–12 wt.%
45	Vortex treatment on SF solution	~35 minutes	0.1–70 (S)	β-sheet	1.3–5.2 wt.%
27	Electrical (direct current) treatment on SF solution	~3 minutes	~1 (S)	Amorphous	8.4 wt.%
46	Addition of ethylene glycol diglycidyl ether in SF solution at 50 °C	Within 2 hours	0.01–100 (S)	β-sheet	4.2 wt.%
47	Addition of sodium dodecyl sulfate in SF solution	~15 minutes	—	β-sheet	4% (wt/vol)
48	Addition of methylcellulose in SF solution at 50 °C	~40 hours	—	β-sheet	2 wt.%
49	Freezing the SF solution, and then immersion them in ethanol	Overnight	—	β-sheet	5 wt.%
39 b	HRP mediated crosslinking of SF solution	1 hour	0.2–10 (S)	Random coil	1–6 wt.%

Ref.: reference; (C): compressive modulus; (S): storage modulus tested in rheometer.

^a^Aqueous SF solution was mixed with cells (final concentration: 0.5 million/mL). The mixture would gel in 0.5–2 hours. The compression modulus was tested without cells.

^b^The hydrogels was allowed to cure for 1 hour at 37 °C for cell seeding, while the shortest gelling time was not mentioned.
