# Supplementary material for: Tumor Growth Suppression Induced by Biomimetic Silk Fibroin Hydrogels
Source: Sci Rep. 2016 Aug 3;6:31037. doi: 10.1038/srep31037 (PMC4971568; doi:10.1038/srep31037)

Supplementary information

Tumor Growth Suppression Induced by Biomimetic Silk Fibroin Hydrogels

Le-Ping Yan[1,2], Joana Silva-Correia[1,2], Viviana P. Ribeiro[1,2], Vera Miranda-Gonçalves[2,3], Cristina Correia[1,2], Alain da Silva Morais[1,2], Rui A. Sousa[1,2], Rui M. Reis[2,3,4], Ana L. Oliveira[1,2,5], Joaquim M. Oliveira[1,2,*], and Rui L. Reis[1,2]

[1]3B's Research Group–Biomaterials, Biodegradables and Biomimetics, University of Minho, Headquarters of the European Institute of Excellence on Tissue Engineering and Regenerative Medicine, AvePark, Parque de Ciência e Tecnologia, Zona Industrial da Gandra, 4805-017 Barco, Guimarães, Portugal.

[2]ICVS/3B's–PT Government Associate Laboratory, Braga/Guimarães, Portugal.

[3]Life and Health Science Research Institute (ICVS), School of Health Sciences, University of Minho, Campus de Gualtar, 4710-057 Braga, Portugal.

[4]Molecular Oncology Research Center, Barretos Cancer Hospital, Barretos, São Paulo, Brazil.

[5]CBQF–Center for Biotechnology and Fine Chemistry, School of Biotechnology, Portuguese Catholic University, Porto, 4200 – 072, Portugal.

*Corresponding author: Joaquim M. Oliveira

Tel: +351-253-510931 (Direct) or +351-253-510900 (General)

Fax: +351-253-510909

E-mail: miguel.oliveira@dep.uminho.pt

Supplementary Figures

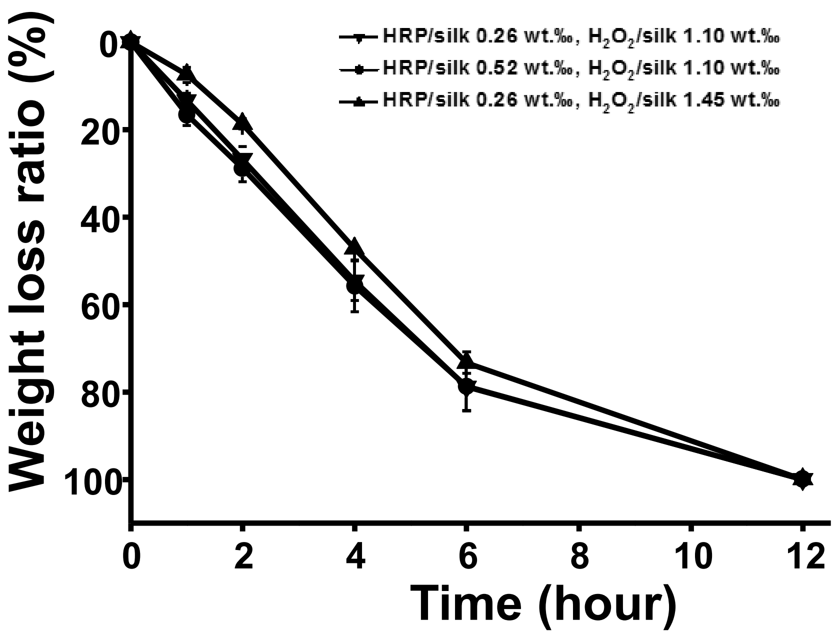

Figure S1. Enzymatic degradation for the SF hydrogels (n=5).

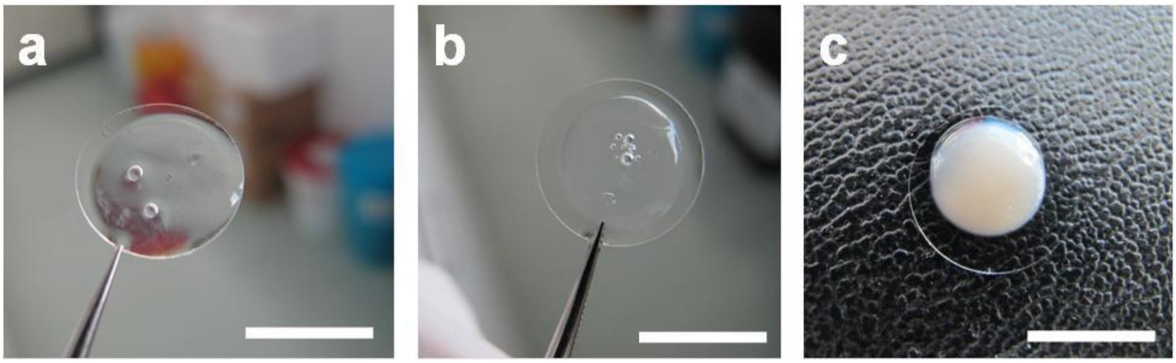

Figure S2. Macroscopic images for the SF hydrogels with ATDC-5 cells encapsulated. (a), (b) and (c): at day 1, day 6 and day 10, respectively. Scale bar: 1 cm.

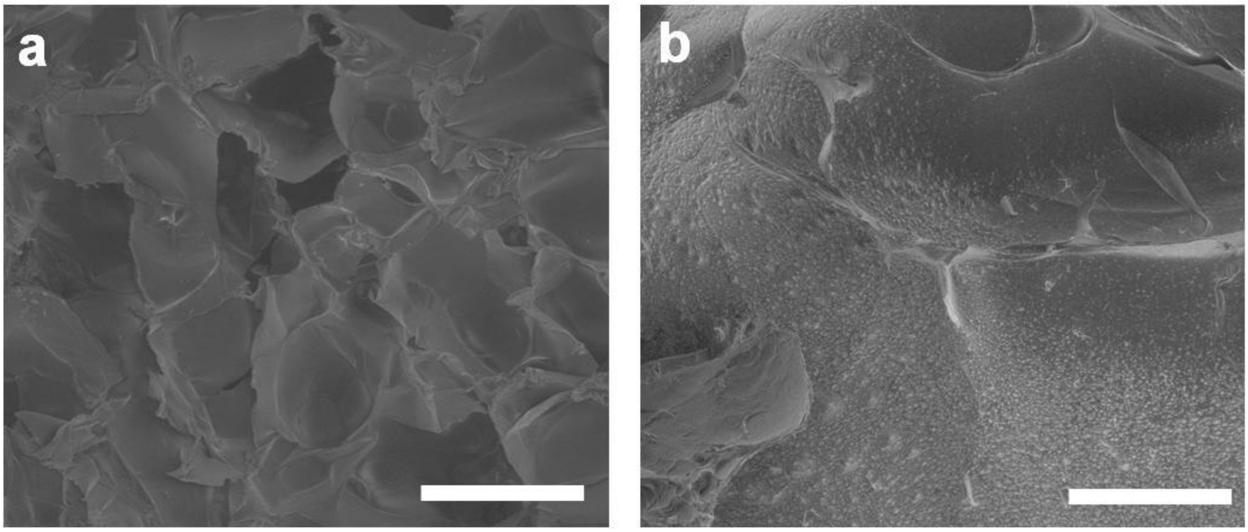

Figure S3. SEM images of the cell encapsulated SF hydrogels. (a), (b): at day 6 and day 10, respectively. Scale bar: 200 μm.

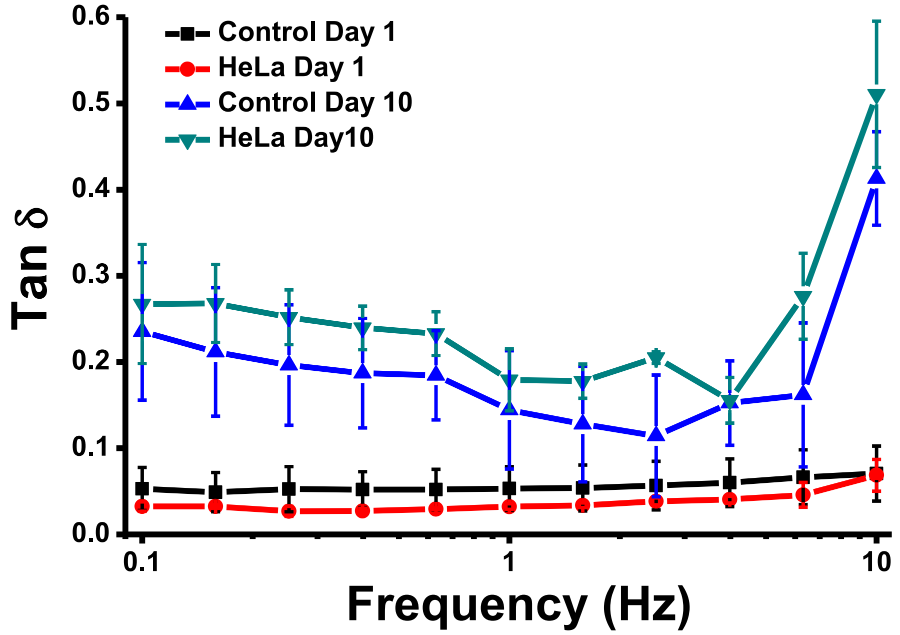

Figure S4. HeLa cells-laden hydrogel loss factor measured by dynamic mechanical analysis. Control Day 1 and Control Day 10: hydrogels without cells encapsulated freshly prepared and ten days later, respectively; HeLa Day 1 and HeLa Day 10: hydrogels encapsulated with HeLa cells for one day and ten days, respectively. (n=3).

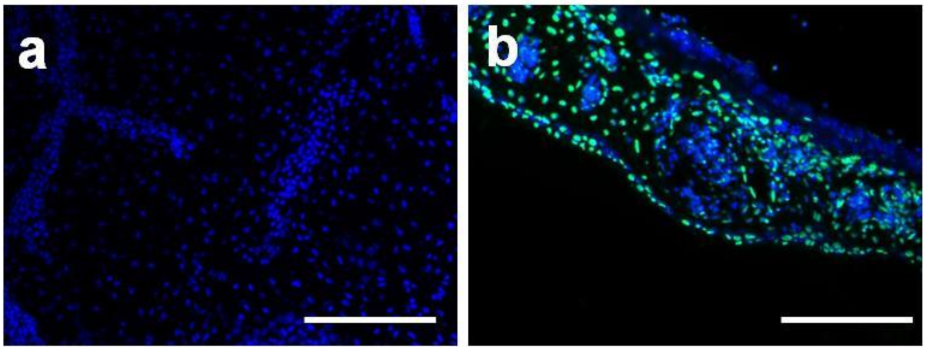

Figure S5. Control for TUNEL assay. (a) Negative (without terminal transferase) and (b) positive controls (with recombinant DNase I). Scale bar: 200 µm.

Table of Content

Silk fibroin hydrogels prepared via peroxidase mediated crosslinking present dominant random coil conformation. These hydrogels irreversibly change to β-sheet conformation after a few days *in vitro* and *in vivo*. HeLa cells can be encapsulated in these hydrogels in random coil status, while the later β-sheet transition of the hydrogel induce cell apoptosis and therefore suppress the tumor formation.

Mechanism of apoptosis of cells in the silk fibroin hydrogels

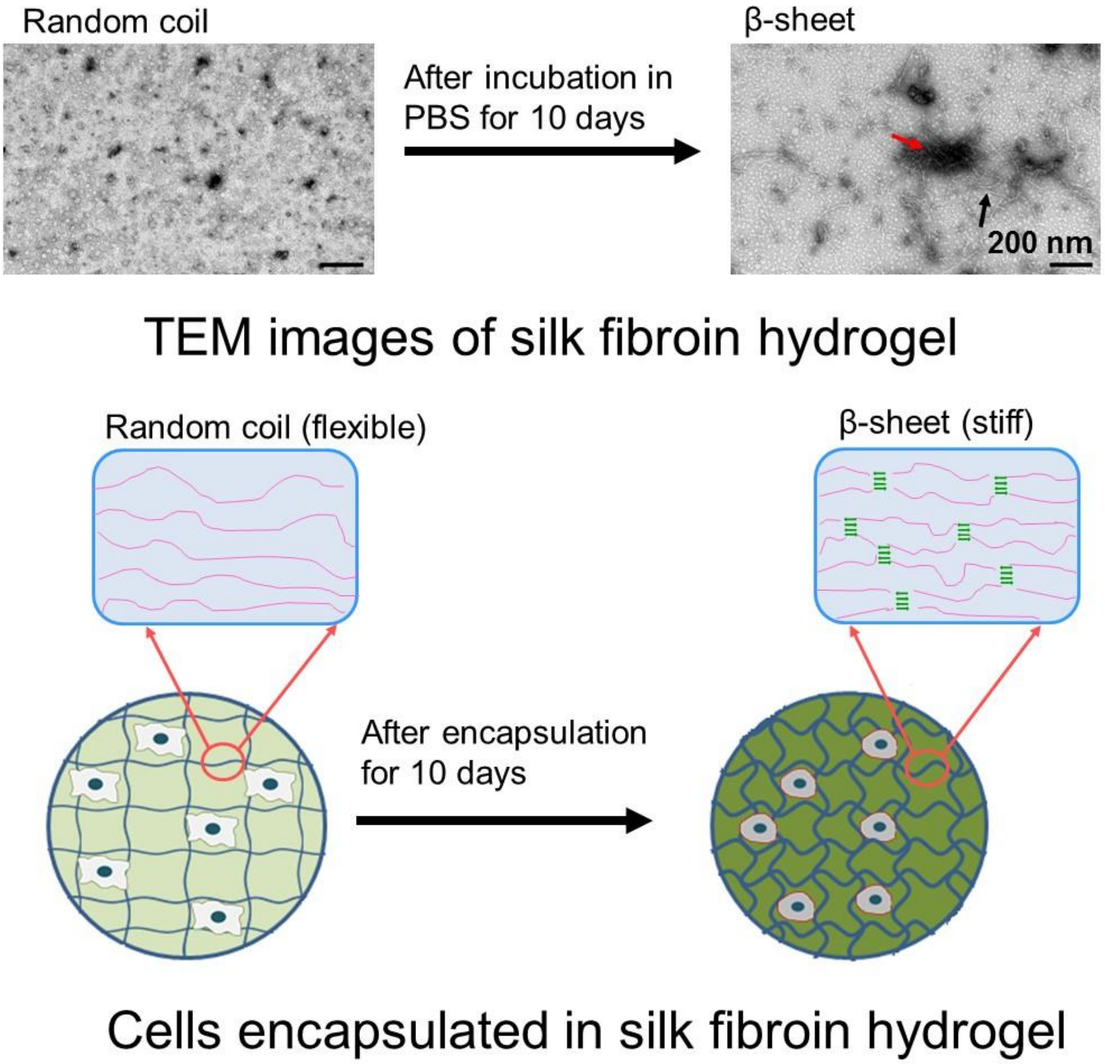

Supplement: Supplementary Information [file srep31037-s1.pdf]
